# Deletion of morpholino binding sites (DeMOBS) to assess specificity of morphant phenotypes

**DOI:** 10.1038/s41598-020-71708-1

**Published:** 2020-09-21

**Authors:** Carlee MacPherson Cunningham, Gianfranco Bellipanni, Raymond Habas, Darius Balciunas

**Affiliations:** Department of Biology, College of Science and Technology, Temple University, Philadelphia, PA 19122 USA

**Keywords:** Developmental biology, Development

## Abstract

Two complimentary approaches are widely used to study gene function in zebrafish: induction of genetic mutations, usually using targeted nucleases such as CRISPR/Cas9, and suppression of gene expression, typically using Morpholino oligomers. Neither method is perfect. Morpholinos (MOs) sometimes produce off-target or toxicity-related effects that can be mistaken for true phenotypes. Conversely, genetic mutants can be subject to compensation, or may fail to yield a null phenotype due to leakiness (e.g. use of cryptic splice sites or downstream AUGs). When discrepancy between mutant and morpholino-induced (morphant) phenotypes is observed, experimental validation of such phenotypes becomes very labor intensive. We have developed a simple genetic method to differentiate between genuine morphant phenotypes and those produced due to off-target effects. We speculated that indels within 5′ untranslated regions would be unlikely to have a significant negative effect on gene expression. Mutations induced within a MO target site would result in a Morpholino-refractive allele thus suppressing true MO phenotypes whilst non-specific phenotypes would remain. We tested this hypothesis on one gene with an exclusively zygotic function, *tbx5a*, and one gene with strong maternal effect, *ctnnb2*. We found that indels within the Morpholino binding site are indeed able to suppress both zygotic and maternal morphant phenotypes. We also observed that the ability of such indels to suppress morpholino phenotypes does depend on the size and the location of the deletion. Nonetheless, mutating the morpholino binding sites in both maternal and zygotic genes can ascertain the specificity of morphant phenotypes.

## Introduction

Methods used to analyze gene loss-of-function fall into two categories: “knockout”, which aims to inactivate the gene of interest by introducing mutations using techniques such as CRISPR/Cas9, and “knockdown”, which aims to abrogate the expression of the gene of interest by employing methods such as siRNA, CRISPRi or Morpholino oligomers (for a recent review, see^1^).

Morpholino oligomers are the most widely used antisense knockdown technology in the zebrafish^2,3^. They inhibit gene expression by blocking either translation or splicing. Translation-blocking MOs base pair with the mRNA either at or upstream of the translation start site and prevent assembly of the 80S ribosome. They inhibit expression of both zygotic and maternally deposited mRNAs and can be used to phenocopy maternal-zygotic mutants. Splice-blocking MOs bind to pre-mRNA at either the splice acceptor or the splice donor site and prevent assembly of the spliceosome, thus abrogating expression of zygotic transcripts but having no effect on maternally deposited mature mRNAs.

MO technology, however, suffers from a significant drawback: off-target effects. In zebrafish, some off-target effects caused by activation of the p53 pathway can be suppressed by co-injection of a standard MO targeting p53^4^. Nonetheless, presence of off-target effects necessitates thorough and labor-intensive validation of morphant phenotypes, including mRNA rescue and use of multiple MOs targeting the gene of interest^5,6^.

Despite these limitations, Morpholino oligomers remain an essential tool to inhibit gene expression in model systems where establishment of genetic mutants is not practically feasible, such the African clawed frog or the chick^7–11^. Certain experimental scenarios will continue to necessitate use of MOs in zebrafish, too. It is the only method that enables generation of large batches gene-inactivated embryos for proteomic, biochemical or imaging analyses^12,13^, especially when analysis is confounded by genetic redundancy^14^.

It is not uncommon for knockdown- and knockout-based approaches to yield different results^15–18^. Such discrepancies have also been observed in zebrafish^19–23^. Sometimes they can be attributed to built-in shortcomings of each approach. Mutant alleles may exhibit leakiness: small indels do not always result in complete loss-of-function due to a variety of phenomena including splicing artifacts and translation initiation at downstream AUGs leading to production of a functional protein and masking the null phenotype^24–26^. A further complication in the analysis of mutant phenotypes arises from the fact that for some genes, maternally-deposited mRNAs (and proteins) partly mask mutant phenotypes necessitating the use of maternal-zygotic mutants^27,28^. Additionally, some frameshift and nonsense mutants induce transcriptional compensation by closely related genes^22,29,30^. Deleting the whole coding sequence appears to be the best way to eliminate these possibilities. However, regulatory complexity of vertebrate genomes raises the possibility that the observed phenotype may be caused by deletion of intron-residing *cis*-regulatory elements for other genes (for an example, see^31,32^).

With the notable exception of short upstream reading frames^33^, 5′ UTRs appear to be sparse in significant regulatory features. We speculated that indel mutations within 5′ UTRs are unlikely to significantly impair the expression of the downstream gene^34^. Indels introduced within a morpholino target site should reduce, if not entirely abolish, MO binding making the “mutant mRNA” partly or completely refractive to morpholino activity. We further hypothesized that since few genes are haploinsufficient, heterozygosity for such MO-refractive mutations would be sufficient to suppress specific morpholino phenotypes. Using *tbx5a* and *ctnnb2* as test loci, we demonstrate that deletions can be readily generated and used to test the specificity of both zygotic and maternal morphant phenotypes.

## Results and discussion

### Partial suppression of *tbx5a* morphant phenotypes by the (− 7) mutation in MO target site

**Figure 1:**
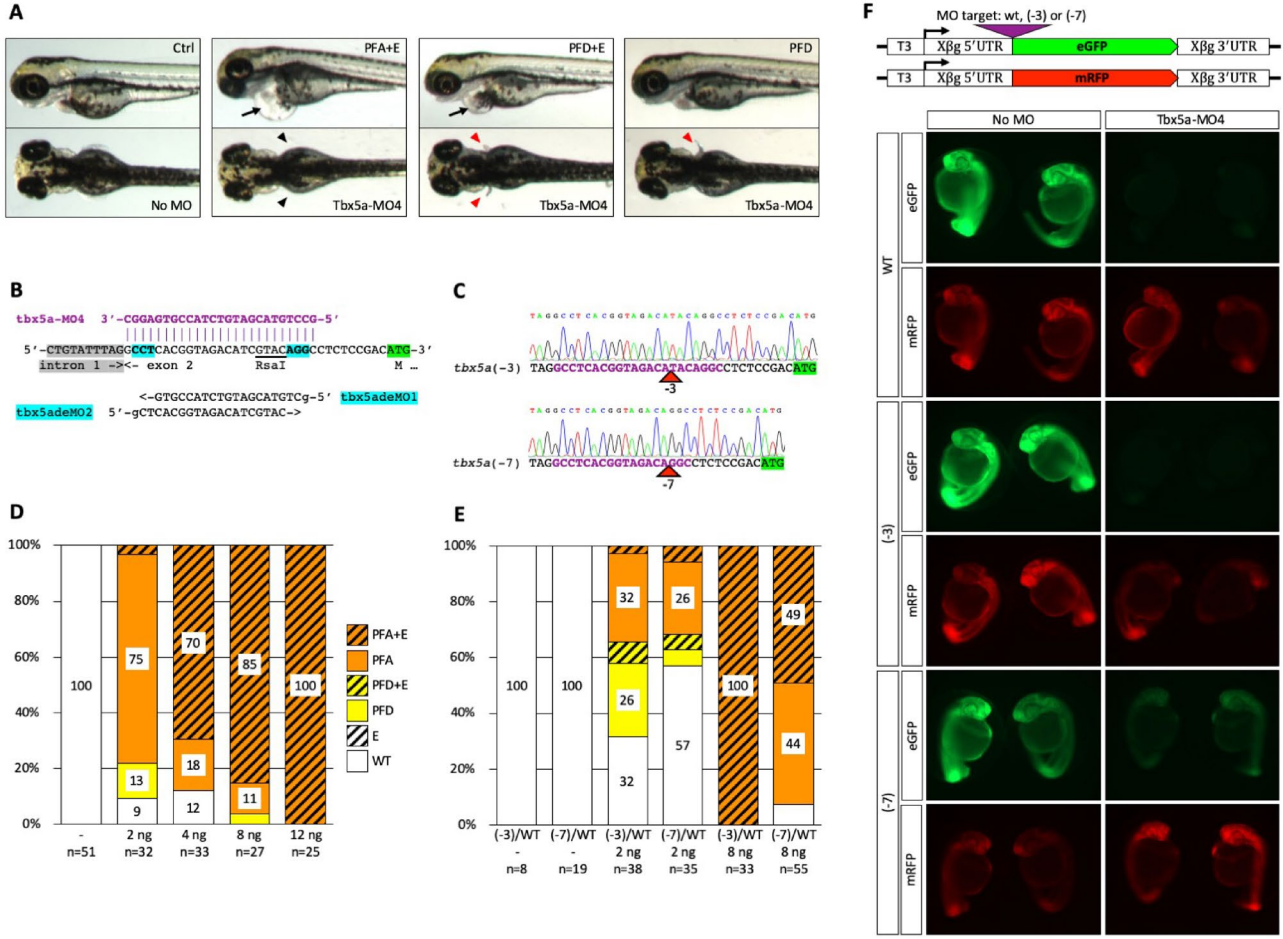
Partial rescue of Tbx5a-MO4 morphant phenotype by (− 3) and (− 7) binding site mutations. (**A**) Embryos injected with Tbx5a-MO4 display a range of *tbx5a* loss of function phenotypes including pectoral fin malformation or absence and severe cardiac edema. Black arrow denotes cardiac edema, black arrowheads denote pectoral fin loss, red arrowheads denote pectoral fin defects. (PFA + E, pectoral fins absent with edema, PFD + E, pectoral fin defect with edema, PFD, pectoral fin defect only) (**B**) Two sgRNAs, tbx5adeMO1 and tbx5adeMO2 targeting the tbx5a-MO4 binding site (MO sequence shown above in purple, PAM sites are highlighted in magenta, coding sequence is highlighted in green. Tbx5adeMO2 overlaps a RsaI restriction enzyme site used for genotyping. (**C**) Sequence confirmation of the (− 3) and (− 7) deletion alleles. (**D**) Titration of Tbx5a-MO4 in wild type (TFL) embryos. Numbers indicate percentages of embryos displaying designated morphant phenotypes. WT is wild-type, E is edema only. (**E**) Suppression of cardiac and/or pectoral fin phenotypes by the (− 3) and (− 7) binding site deletions at different doses of MO. Adults heterozygous for either the (− 3) or (− 7) deletion were outcrossed and embryos were injected with either 2 ng or 8 ng of tbx5a-MO4. (F) Tbx5a-MO4 is able to at least partly block the translation of mRNAs containing (− 3) and (− 7) binding site mutations. In vitro transcribed mRNAs from each eGFP construct was injected along with mRFP mRNA as a control, and half of the mRNA-injected embryos were then injected with tbx5a-MO4. At 1dpf, embryos displaying similar levels of mRFP expression were photographed. T3, T3 transcription start site, Xβg 5′ UTR and Xβg 3′ UTR, Xenopus β-globin 5′ and 3′ untranslated regions, respectively.

Tbx5a mutants and morphants display absent or malformed pectoral fins and a linear heart Fig. 1A^35–38^. Since *tbx5a* mRNA is not contributed maternally, outcross of a parent heterozygous for a potentially MO-refractive mutation would produce a clutch of embryos where half would be genotypically wild type and susceptible to the MO, while the other half would be refractive to the MO. Susceptible and refractive embryos, present within a single clutch, would serve as controls for each other, eliminating experimental bias by excluding variables such as active MO concentration, injection volume or timing of the injection.

Two *S. pyogenes* PAM (protospacer adjacent motif) sites are present within the 5′ UTR sequence targeted by tbx5a-MO4^39^ (Fig. 1B, Supplementary Table 1). We synthesized 19-base guide RNAs with a G nucleotide (lower case in Fig. 1B, Supplementary Table 2) required for transcription initiation by the T7 RNA polymerase. Guide RNAs were injected along with nCas9n mRNA as previously described^34,40^. PCR fragments amplified from lysates of 20 pooled 3 day post fertilization (dpf) injected embryos were analyzed for sgRNA efficiency by TIDE^41^ and Synthego ICE^42^. Both analyses showed that tbx5adeMO2 sgRNA (~ 30% by TIDE, ~ 11% by ICE) was more efficient than tbx5adeMO1 sgRNA (~ 17% by TIDE, ~ 7% by ICE) (Supplementary Fig. 1). We raised embryos injected with tbx5adeMO2 sgRNA and nCas9n and screened three F0 fish for germline transmission of indels using the T7 endonuclease assay (data not shown). One founder produced a high percentage of progeny with indels, and one F1 family was raised. Four out of seven genotyped adult F1s were found to be heterozygous for indels: two for a (− 3) deletion and two for a (− 7) deletion by Poly Peak Parser^43^ analysis. F1s heterozygous for (− 3) and (− 7) deletions were incrossed. All embryos were phenotypically normal, indicating that these deletions do not significantly impair the expression of *tbx5a*. Sequence of the (− 3) and (− 7) deletions was confirmed on homozygous F2s (Fig. 1C).

To determine the effective dose of Tbx5a-MO4, we injected 2, 4, 8 and 12 ng of the morpholino into one-cell zebrafish embryos (Fig. 1D). The lowest dose of the MO resulted in > 90% of embryos displaying pectoral fin defects. In contrast, a much higher 8 ng dose of the MO was needed to elicit severe cardiac defects (> 90% edema). Notably, in humans suffering from Holt–Oram syndrome caused by mutations in Tbx5, forelimb defects are also more penetrant and severe than cardiac defects^44–46^.

We outcrossed F1 fish heterozygous for the (− 3) and (− 7) deletions and injected embryos with 2 ng or 8 ng Tbx5a-MO4. In each cross, approximately 50% of embryos were expected to be genetically wild type and therefore display morphant phenotypes, while the other 50% were expected to inherit the corresponding deletion, leading to either partial or complete suppression of MO phenotypes. At the low 2 ng MO dose, 12/38 (32%) of embryos from the (− 3) heterozygote outcross were phenotypically wild type, whilst 10/38 (26%) showed the milder pectoral fin defect phenotype, indicating full or partial rescue in approximately 50% of the progeny as expected. Among embryos from the (− 7) heterozygote outcross, 20/35 (57%) showed full rescue of both the cardiac edema and pectoral fin loss phenotype (Fig. 1E). Embryos from both outcrosses were grouped by phenotype and genotyped. In the (− 3) outcross, genotyping revealed that 6/8 (75%) embryos displaying pectoral fin loss or defects were wild-type, and 7/8 (88%) phenotypically wild-type embryos were heterozygous for the (− 3) deletion (*P* = 0.015). In the (− 7) outcross, genotyping revealed that 8/8 (100%) embryos displaying pectoral fin loss or defects were wild-type, and 7/8 (88%) phenotypically wild-type embryos were heterozygous for the (− 7) deletion (*P* = 0.002). These results confirmed that at a low dose of the morpholino, the (− 3) allele can largely suppress the morphant phenotype while the (− 7) allele shows complete rescue.

At the high 8 ng MO dose, all MO-injected embryos from the (− 3) outcrosses displayed identical morphant phenotypes (Fig. 1E). In contrast, approximately 50% of embryos from the (− 7) outcross were completely rescued from the cardiac edema phenotype, but not the pectoral fin defect (Fig. 1E). Embryos from the (− 7) outcross were grouped by phenotype and subsequently genotyped. Genotyping revealed that 14/15 (93%) of individuals with cardiac edema and loss of pectoral fins were wild-type and 13/16 (81%) of individuals with no cardiac edema were heterozygous for the (− 7) allele (*P* = 3.0E−05) (Supplementary Fig. 2). These results indicate rescue of the cardiac edema morphant phenotype, but not the pectoral fin phenotype, by heterozygosity for the (− 7) allele at the high dose of the Morpholino.

**Figure 2:**
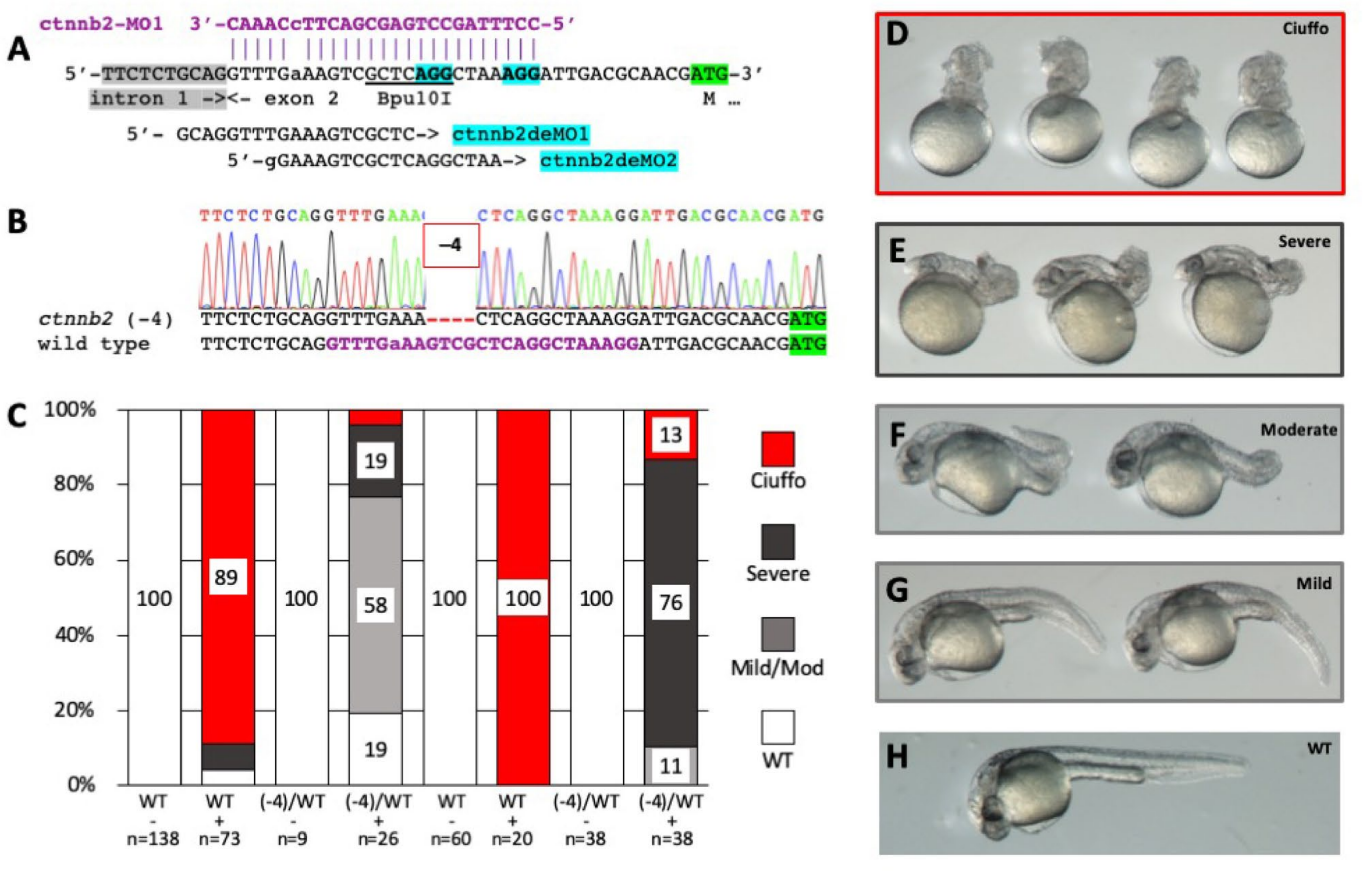
Suppression of a maternal morphant phenotype by DeMOBS. (**A**) Two sgRNAs, ctnnb2deMO1 and ctnnb2deMO2 target ctnnb2-MO1 binding site. ctnnb2deMO1 overlaps a Bpu10I restriction enzyme site used for genotyping. A single nucleotide polymorphism resulting in a single base mismatch between the MO and the target sequence is shown in lower case. (**B**) Confirmation of the (− 4) allele by sequence analysis. (**C**) Co-injection ctnnb2-MO1 and ctnnb1-MO2 into embryos obtained from four female siblings (two wild type and two heterozygous for the deletion) in blind experiments. (**D**) *ciuffo* morphant phenotype resulting from injection of ctnnb1-MO2 and ctnnb2-MO1. (**E**–**H**) Residual phenotypes observed in MO-injected embryos from a female heterozygous for the MO-refractive (− 4) mutation.

Introduction of a MO-refractive mutation into mRNA has the potential to exacerbate off-target effects. In heterozygous embryos, only 50% of target mRNA can be bound by the MO, thus increasing the concentration of morpholino available for off-target binding. The fact that we did not observe any new phenotypes in heterozygous embryos further supports the observation that Tbx5a-MO4 is highly specific.

Dose- , phenotype- and deletion size-dependent rescue of morphant phenotypes prompted us to hypothesize that (− 3) and (− 7) deletions were insufficient to make mRNAs entirely refractive to the morpholino. We cloned wild type, (− 3) and (− 7) target sites into the pT3TS in vitro transcription vector^47^ ahead of eGFP coding sequence. In vitro transcribed mRNAs were injected into embryos along with pT3TS:mRFP mRNA as a control not affected by the MO. Half of the mRNA-injected embryos were then injected with 8 ng of Tbx5a-MO4. Embryos were scored for RFP and GFP fluorescence and photographed at 1 dpf (Fig. 1F). We found that Tbx5a-MO4 was able to almost entirely block translation of mRNAs containing wild type and (− 3) target sites. Translation of mRNA containing the (− 7) target site was also reduced significantly (Fig. 1F). These findings raise significant concerns about MO specificity. The (− 3) and (− 7) deletions preserved a 16-nucleotide and 15-nucleotide identity to the target site at the 3′ end of the MO (toward the 5′ end of the mRNA), respectively. Our data therefore indicate that a 3′ stretch of identity as short 15–16 nucleotides may be sufficient for a MO to impede translation at a high dose, and suggests that using MOs shorter than the current 25 nucleotide standard may lead to higher specificity.

### Suppression of the β-*catenin* morphant phenotype by maternal contribution of (− 4) binding site mutant *ctnnb2* mRNA

We speculated that for maternally contributed genes, a female heterozygous for a MO-refractive allele would produce embryos which would be refractive to the maternal-zygotic phenotype. To test this hypothesis, we selected beta-catenin genes coding for an essential component of the Wnt signaling pathway. Co-injection of translation-blocking MOs targeting the duplicated *ctnnb1* and *ctnnb2* mRNAs results in complete loss of ventral cell fates and a phenotype named *ciuffo* (Supplementary Table 1)^48^. Partial sequencing of *ctnnb2* loci in the TLF genetic background revealed presence of a single nucleotide polymorphism within the ctnnb2-MO1 binding site. Co-injection of ctnnb1-MO2 and ctnnb2-MO1 into TLF embryos at concentrations described previously^48^ resulted in nearly 100% penetrant *ciuffo* phenotype (data not shown), indicating that the polymorphism alone does not appreciably reduce morpholino activity.

Two sgRNAs targeting PAM sequences within the ctnnb2-MO1 binding site were designed and tested (Fig. 2A, Supplementary Table 2). Only ctnnb2deMO1 had detectable activity by both TIDE (~ 17%) and Synthego ICE (~ 5%) analysis (Supplementary Fig. 3). Three adult F0 fish injected with ctnnb2deMO1 sgRNA and nCas9n mRNA were tested for germline transmission of indels, leading to establishment of one F1 family. Fourteen F1 fish were tested for loss of Bpu10I restriction enzyme site, and seven were found to be heterozygous for indels. PCR fragments from five F1s were sequenced, and four were found to be heterozygous for a (− 4) deletion (Fig. 2B). A male heterozygous for the (− 4) deletion was outcrossed to establish an F2 family.

To avoid experimental bias, we performed a blind experiment to test for suppression of *ciuffo* phenotype. From a single F2 family, we identified two adult females heterozygous for the deletion and two female wild type siblings. Fish were coded A, B, C and D, and embryos obtained from outcrosses were injected with a mixture of ctnnb1-MO2 and ctnnb2-MO1. MO injection lead to high penetrance (89% and 100%) of *ciuffo* phenotypes in the progeny of wild type fish, while presence of one (− 4) allele in heterozygotes almost completely suppressed the *ciuffo* phenotype (4% and 13%, Fig. 2C). Milder phenotypes were still observed in a subset of embryos (Fig. 2D–H), which could reflect zygotic requirement for *ctnnb1* and/or *ctnnb2*^49,50^, or off-target effects due to a high cumulative dose of the two MOs. Nonetheless, we observed that females heterozygous for a (− 4) deletion in combination with a serendipitous single nucleotide polymorphism produce embryos which are nearly 100% suppressed for the *ciuffo* phenotype. These results indicate that our method can be used to ascertain morphant phenotypes of genes with strong maternal contribution of mRNA.

Our data clearly demonstrates that indels within MO binding sites can be readily generated and used to test the specificity of both zygotic and maternal morphant phenotypes. The ability to induce deletions within MO binding sites using CRISPR/Cas9 relies on the presence of a PAM site within the MO binding site, preferably close to the 5′ end of the target site. While the mutagenesis method employed by us is likely feasible for the majority of MOs targeting 5′ UTRs, there will inevitably be a subset where PAM sites will be absent or located closer to the middle or the 3′ of the target site. In such cases, oligonucleotide-mediated repair of double strand breaks can be used to engineer desired mutations^34,40,51–56^.

Inconsistency between knock-out and knock-down phenotypes poses a significant challenge for researchers studying gene function. This inconsistency can be attributed to morpholino off-target effects, incomplete loss-of-function in the knockout, genetic (transcriptional) compensation, or a combination of these factors. For scenarios where the observed knockdown phenotypes are more severe than those seen in genetic mutants, our method offers a fairly quick and cost-effective way to test if the morphant phenotypes are indeed specific.

We selected *tbx5a* and *ctnnb2* for this proof-of-principle study due high degree of confidence we had in respective mutant and morphant phenotypes. These two genes can be taken as models for a broad spectrum of zebrafish genes: tbx5a has an exclusively zygotic function while ctnnb2 mRNA is maternally contributed. Furthermore, *ctnnb2* is functionally duplicated necessitating simultaneous knockdown of *ctnnb1* in order to observe a phenotype. The ability to rescue both *tbx5a* and *ctnnb2* morphants offers a level of confidence that this method can be used for a large subset of other genes that are not as well-characterized.

## Materials and methods

### CRISPR/Cas9 mutagenesis

Guide RNAs were produced as previously described^34,40^ using DR274^57^ as the template and diluted to ~ 60 ng/μL. Immediately prior to injection, 8 uL of diluted sgRNA was mixed with 2 μL aliquot of 150 ng/μL nCas9n mRNA^58^ to the final volume of 10 μL.

### Plasmid construction and mRNA synthesis

Details of plasmid construction are available upon request. eGFP-containing pT3TS^47^ vectors [pCMC23 (wt MO binding site), pCMC24 (− 3) and pCMC25 (− 7)] were linearized using XbaI restriction enzyme. Template for the synthesis of mRFP mRNA was amplified by PCR using M13F/M13R primer pair on pT3TS:mRFP (pDB935). Templates were transcribed using T3 mMessage mMachine kit and mRNAs were purified using Qiagen RNeasy MinElute kit. mRNAs were diluted so that the standard 3 nL injection volume would contain 50 ng of tbx5a-eGFP mRNA and 100 ng of mRFP mRNA.

### Microinjection

Microinjection volumes were calibrated to 3 nL as previously described, and all microinjections were performed into the yolks of 1-cell zebrafish embryos as described^59^.

### Ethics approval

All experiments described in this manuscript have been carried out in accordance with all relevant guidelines and recommendations. Experiments with vertebrate animals have been approved by Temple University Institutional Animal Care and Use Committee (IACUC), ACUP#4709 (PI: Balciunas) and ACUP #4674 (PI: Bellipanni).

## Supplementary information

Supplementary legends.

Supplementary information.

## References

[1] Housden BE (2017). Loss-of-function genetic tools for animal models: Cross-species and cross-platform differences. Nat. Rev. Genet..

[2] Nasevicius A, Ekker SC (2000). Effective targeted gene 'knockdown' in zebrafish. Nat. Genet..

[3] Ekker SC, Larson JD (2001). Morphant technology in model developmental systems. Genesis.

[4] Robu ME (2007). p53 activation by knockdown technologies. PLoS Genet..

[5] Eisen JS, Smith JC (2008). Controlling morpholino experiments: Don't stop making antisense. Development.

[6] Stainier DYR (2017). Guidelines for morpholino use in zebrafish. PLoS Genet..

[7] Blum M, De Robertis EM, Wallingford JB, Niehrs C (2015). Morpholinos: Antisense and sensibility. Dev. Cell.

[8] Gammill LS, Jacques-Fricke B, Roffers-Agarwal J (1920). Embryological and genetic manipulation of chick development. Methods Mol. Biol..

[9] McLennan R, Kulesa PM (1976). In Ovo electroporation of plasmid DNA and morpholinos into specific tissues during early embryogenesis. Methods Mol. Biol..

[10] Ding Y (2018). Bighead is a Wnt antagonist secreted by the Xenopus Spemann organizer that promotes Lrp6 endocytosis. Proc. Natl. Acad. Sci. USA.

[11] Bestman JE, Cline HT (2020). Morpholino studies in xenopus brain development. Methods Mol. Biol..

[12] Zeituni EM, Farber SA (2016). Studying lipid metabolism and transport during zebrafish development. Methods Mol. Biol..

[13] Hashimoto Y, Greco TM, Cristea IM (2019). Contribution of mass spectrometry-based proteomics to discoveries in developmental biology. Adv. Exp. Med. Biol..

[14] Zinski J (2017). Systems biology derived source-sink mechanism of BMP gradient formation. Elife.

[15] Evers B (2016). CRISPR knockout screening outperforms shRNA and CRISPRi in identifying essential genes. Nat. Biotechnol..

[16] Morgens DW, Deans RM, Li A, Bassik MC (2016). Systematic comparison of CRISPR/Cas9 and RNAi screens for essential genes. Nat. Biotechnol..

[17] Luttrell LM (2018). Manifold roles of beta-arrestins in GPCR signaling elucidated with siRNA and CRISPR/Cas9. Sci. Signal.

[18] Bachas C (2018). Rscreenorm: Normalization of CRISPR and siRNA screen data for more reproducible hit selection. BMC Bioinform..

[19] Law SH, Sargent TD (2014). The serine-threonine protein kinase PAK4 is dispensable in zebrafish: Identification of a morpholino-generated pseudophenotype. PLoS ONE.

[20] Novodvorsky P (2015). klf2ash317 mutant zebrafish do not recapitulate morpholino-induced vascular and haematopoietic phenotypes. PLoS ONE.

[21] Kok FO (2015). Reverse genetic screening reveals poor correlation between morpholino-induced and mutant phenotypes in zebrafish. Dev. Cell.

[22] Rossi A (2015). Genetic compensation induced by deleterious mutations but not gene knockdowns. Nature.

[23] Joris M (2017). Number of inadvertent RNA targets for morpholino knockdown in *Danio rerio* is largely underestimated: evidence from the study of Ser/Arg-rich splicing factors. Nucleic Acids Res..

[24] Anderson JL (2017). mRNA processing in mutant zebrafish lines generated by chemical and CRISPR-mediated mutagenesis produces unexpected transcripts that escape nonsense-mediated decay. PLoS Genet..

[25] Lalonde S (2017). Frameshift indels introduced by genome editing can lead to in-frame exon skipping. PLoS ONE.

[26] Smits AH (2019). Biological plasticity rescues target activity in CRISPR knock outs. Nat. Methods.

[27] Gritsman K (1999). The EGF-CFC protein one-eyed pinhead is essential for nodal signaling. Cell.

[28] Miller-Bertoglio V (1999). Maternal and zygotic activity of the zebrafish ogon locus antagonizes BMP signaling. Dev. Biol..

[29] El-Brolosy MA (2019). Genetic compensation triggered by mutant mRNA degradation. Nature.

[30] Ma Z (2019). PTC-bearing mRNA elicits a genetic compensation response via Upf3a and COMPASS components. Nature.

[31] Zhou F, Leder P, Zuniga A, Dettenhofer M (2009). Formin1 disruption confers oligodactylism and alters Bmp signaling. Hum. Mol. Genet..

[32] Zuniga A (2012). Conserved cis-regulatory regions in a large genomic landscape control SHH and BMP-regulated Gremlin1 expression in mouse limb buds. BMC Dev. Biol..

[33] Johnstone TG, Bazzini AA, Giraldez AJ (2016). Upstream ORFs are prevalent translational repressors in vertebrates. EMBO J..

[34] Burg L (2018). Conditional mutagenesis by oligonucleotide-mediated integration of loxP sites in zebrafish. PLoS Genet..

[35] Ahn DG, Kourakis MJ, Rohde LA, Silver LM, Ho RK (2002). T-box gene tbx5 is essential for formation of the pectoral limb bud. Nature.

[36] Ng JK (2002). The limb identity gene Tbx5 promotes limb initiation by interacting with Wnt2b and Fgf10. Development.

[37] Garrity DM, Childs S, Fishman MC (2002). The heartstrings mutation in zebrafish causes heart/fin Tbx5 deficiency syndrome. Development.

[38] Grajevskaja V, Camerota D, Bellipanni G, Balciuniene J, Balciunas D (2018). Analysis of a conditional gene trap reveals that tbx5a is required for heart regeneration in zebrafish. PLoS ONE.

[39] Lu JH (2008). Cascade effect of cardiac myogenesis gene expression during cardiac looping in tbx5 knockdown zebrafish embryos. J. Biomed. Sci..

[40] Burg L (2016). Internal epitope tagging informed by relative lack of sequence conservation. Sci. Rep..

[41] Brinkman EK, Chen T, Amendola M, van Steensel B (2014). Easy quantitative assessment of genome editing by sequence trace decomposition. Nucleic Acids Res..

[42] Hsiau, T. *et al.* Inference of CRISPR edits from sanger trace data. *bioRxiv*. https://www.biorxiv.org/content/10.1101/251082v1 (2019). 10.1089/crispr.2021.0113 35119294

[43] Hill JT (2014). Poly peak parser: Method and software for identification of unknown indels using sanger sequencing of polymerase chain reaction products. Dev. Dyn..

[44] Smith AT, Sack GH, Taylor GJ (1979). Holt–Oram syndrome. J. Pediatr..

[45] Basson CT (1994). The clinical and genetic spectrum of the Holt–Oram syndrome (heart-hand syndrome). N. Engl. J. Med..

[46] Basson CT (1997). Mutations in human TBX5 [corrected] cause limb and cardiac malformation in Holt–Oram syndrome. Nat. Genet..

[47] Hyatt TM, Ekker SC (1999). Vectors and techniques for ectopic gene expression in zebrafish. Methods Cell Biol..

[48] Bellipanni G (2006). Essential and opposing roles of zebrafish beta-catenins in the formation of dorsal axial structures and neurectoderm. Development.

[49] Varga M, Maegawa S, Bellipanni G, Weinberg ES (2007). Chordin expression, mediated by Nodal and FGF signaling, is restricted by redundant function of two beta-catenins in the zebrafish embryo. Mech. Dev..

[50] Valenti F (2015). The increase in maternal expression of axin1 and axin2 contribute to the zebrafish mutant ichabod ventralized phenotype. J. Cell Biochem..

[51] Dong Z, Dong X, Jia W, Cao S, Zhao Q (2014). Improving the efficiency for generation of genome-edited zebrafish by labeling primordial germ cells. Int. J. Biochem. Cell Biol..

[52] Hruscha A (2013). Efficient CRISPR/Cas9 genome editing with low off-target effects in zebrafish. Development.

[53] Bedell VM (2012). In vivo genome editing using a high-efficiency TALEN system. Nature.

[54] Gagnon JA (2014). Efficient mutagenesis by Cas9 protein-mediated oligonucleotide insertion and large-scale assessment of single-guide RNAs. PLoS ONE.

[55] Prykhozhij SV (2018). Optimized knock-in of point mutations in zebrafish using CRISPR/Cas9. Nucleic Acids Res..

[56] Gibb N (2018). Hey2 regulates the size of the cardiac progenitor pool during vertebrate heart development. Development.

[57] Hwang WY (2013). Efficient genome editing in zebrafish using a CRISPR-Cas system. Nat. Biotechnol..

[58] Jao LE, Wente SR, Chen WB (2013). Efficient multiplex biallelic zebrafish genome editing using a CRISPR nuclease system. Proc. Natl. Acad. Sci. USA.

[59] Balciuniene J, Balciunas D (2013). Gene trapping using gal4 in zebrafish. J. Vis. Exp..

