# Supplementary material for: Deletion of morpholino binding sites (DeMOBS) to assess specificity of morphant phenotypes
Source: Sci Rep. 2020 Sep 21;10:15366. doi: 10.1038/s41598-020-71708-1 (PMC7506532; doi:10.1038/s41598-020-71708-1)
Supplement: Supplementary file 1 — Supplementary legends. [file 41598_2020_71708_MOESM1_ESM.pdf]

Supp. Figure 1. Testing the efficiency tbx5adeMO1 or tbx5adeMO2 sgRNAs by sequencing. Targeted locus was amplified with primers tbx5ain1-F2 and tbx5aex2-R2, using lysate of 20 pooled embryos as the template. PCR fragments were sequenced using tbx5aex2-R2. (A) Sequencing of embryos injected with tbx5adeMO1. Please note that reverse complement of sequencing chromatogram is provided to correspond to Figure 1B. The expected location of the double strand break is indicated by the red arrow. (B) Sequencing of embryos injected with tbx5adeMO1. (C) Efficiency of the two guides and most prevalent deletions as estimated by TIDE (https://tide.deskgen.com/) and Synthego ICE (https://ice.synthego.com/#/).

Supp. Figure 2. Genotyping of Tbx5a-MO4-injected embryos for the *tbx5a* (-7) deletion. (A) Diagram of the PCR fragment and restriction enzyme digest. Black arrows indicate the primers used for PCR amplification. Dashed lines indicate RsaI restriction enzyme sites. Blue lines show digestion products indicative of the wild type allele, red line - digestion product indicative of the (-7) allele. (B) Genotyping of individual 3 dpf embryos scored as positive (#1-16) or negative (#17-32) for cardiac edema. Sample numbers in red indicate that the embryo was scored as a heterozygote. Sample #15 was deemed inconclusive. GeneRuler DNA Ladder (ThermoFisher Scientific) is used for size comparison.

Supp. Figure 3. Testing the efficiency Testing the efficiency ctnnb2deMO1 or ctnnb2deMO2 guide RNAs by sequencing. Targeted locus was amplified with primers ctnnb2-F1 and ctnnb2-R1, using lysate of 20 pooled embryos as the template, and the obtained PCR fragments were sequenced using ctnnb2-F1. (A) Sequencing of embryos injected with ctnnb2deMO1. The expected location of the double strand break is indicated by the red arrow. Sequencing of embryos injected with ctnnb2deMO2. (C) Efficiency of the two guides and most prevalent deletions as estimated by TIDE (https://tide.deskgen.com/) and Synthego ICE (https://ice.synthego.com/#/).

Supplementary Table 1. Primer sequences

Supplementary Table 2. Sequences of Morpholino oligomers.
