# Supplementary material for: Deletion of morpholino binding sites (DeMOBS) to assess specificity of morphant phenotypes
Source: Sci Rep. 2020 Sep 21;10:15366. doi: 10.1038/s41598-020-71708-1 (PMC7506532; doi:10.1038/s41598-020-71708-1)
Supplement: Supplementary file 2 — Supplementary information. [file 41598_2020_71708_MOESM2_ESM.pdf]

A

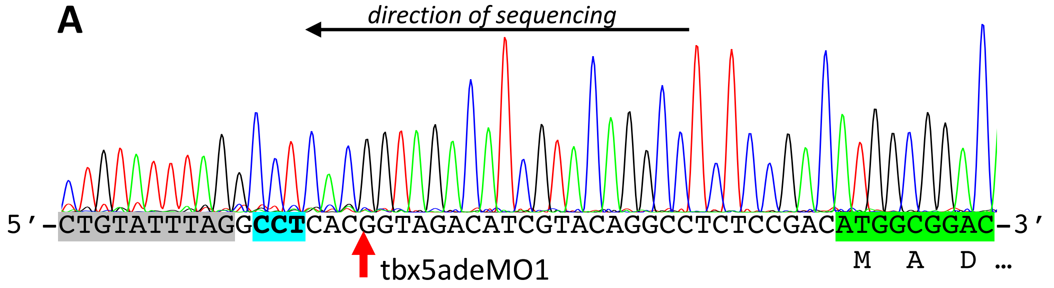

B

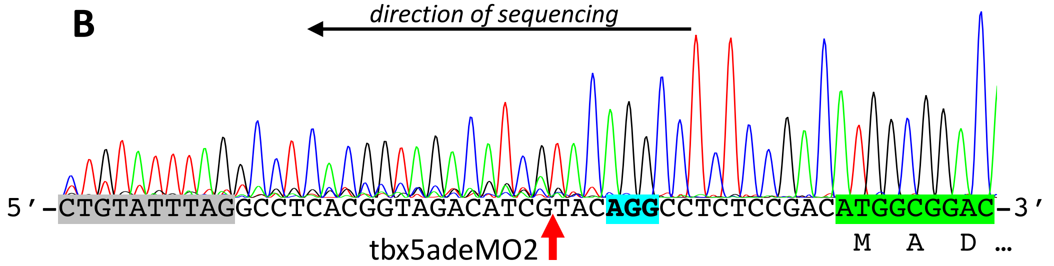

C

	TIDE efficiency score	TIDE most prominent indels	ICE efficiency score	ICE most prominent indels
**tbx5adeMO1**	16.9%	(+1) 5.2%, (-7) 3.0%, (-1) 2.4%	7%	(+1) 4.0%, (-12) 2.0%, (-7), 1.0%
**tbx5adeMO2**	29.9%	(-3) 8.7%, (-7) 6.7%, (-4) 2.8%	11%	(-3) 4.0%, (-7) 3.0%

Supplementary Figure 1

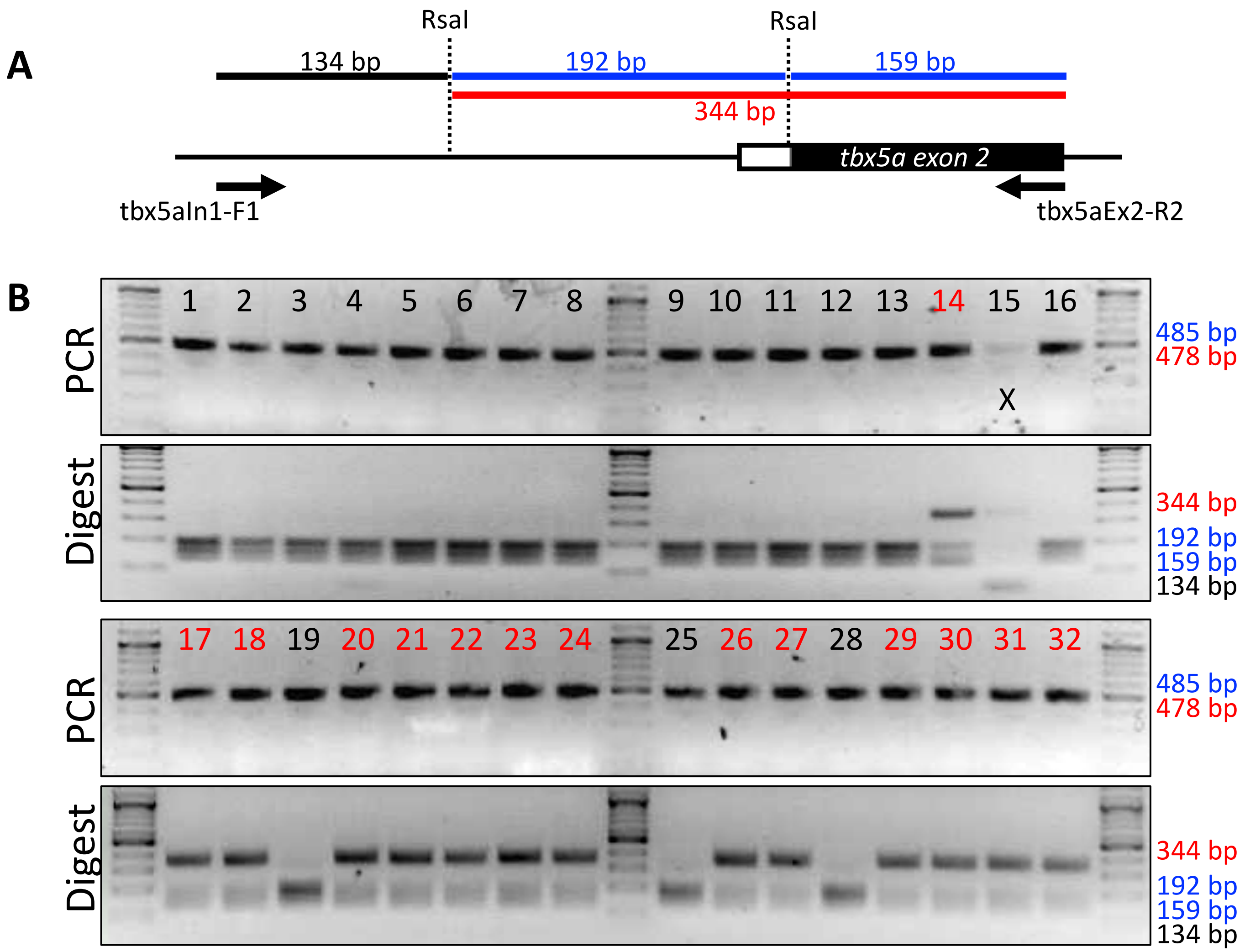

Supplementary Figure 2

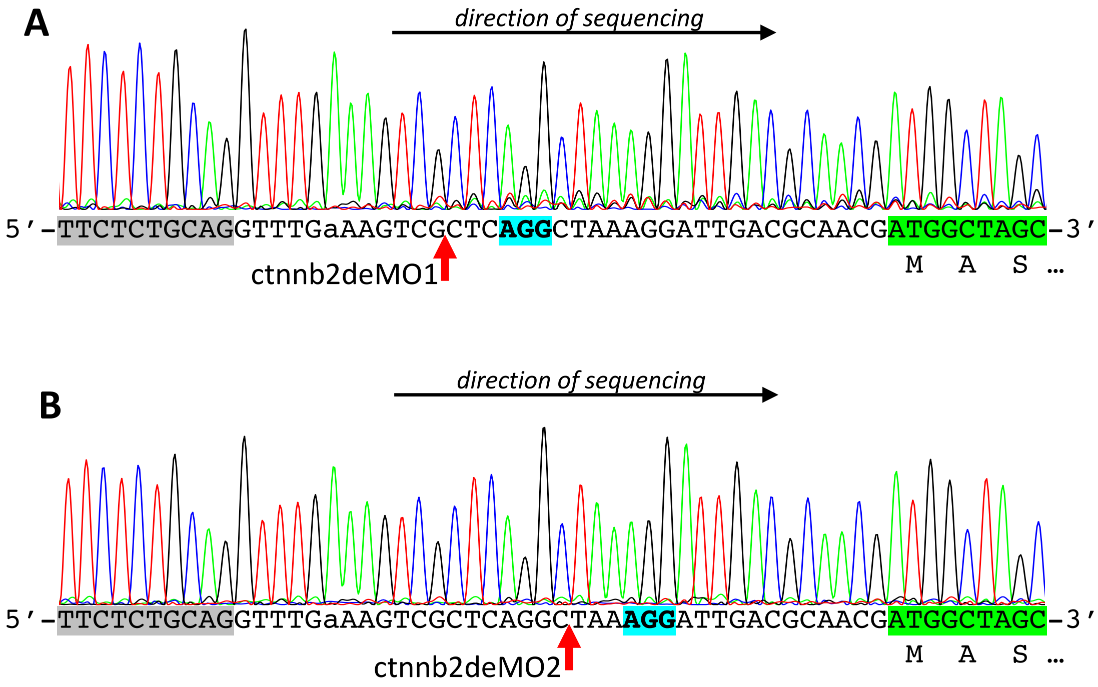

C

	TIDE efficiency score	TIDE most prominent indels	ICE efficiency score	ICE most prominent indels
**ctnnb2 deMO1**	17.2%	(-4) 7.0%, (-5) 2.1%, (-2) 2.0%	5%	(-4) 5.0%
**ctnnb2 deMO2**	4.4%	(-4) 1.3%, (-6) 0.7%, (-8) 0.6%	0%	---

Supplementary Figure 3

Primer	Sequence 5′ to 3′
Genotyping primers	
tbx5aIn1-F2	CAGATTCATGAACTATCGGTGTACA
tbx5aEx2-R2	CTGTTGAATGTATGTAGTCTGCGAT
ctnnb2-F1	CTGGCAATTCCTAATGACTCAGTCT
ctnnb2-R1	AGCCTATAGCGATAAGCTAAATCAC
Specific primers used for guide RNA synthesis	
tbx5adeMO1	CGCTAGCTAATACGACTCACTATA**gCTGTACGATGTCTACCGTG**GTTTTAGAGCTAGAAATAG
tbx5adeMO2	CGCTAGCTAATACGACTCACTATA**gCTCACGGTAGACATCGTAC**GTTTTAGAGCTAGAAATAG
ctnnb2deMO1	CGCTAGCTAATACGACTCACTATA**GCAGGTTTGAAAGTCGCTC**GTTTTAGAGCTAGAAATAG
ctnnb2deMO2	CGCTAGCTAATACGACTCACTATA**gGAAAGTCGCTCAGGCTAA**GTTTTAGAGCTAGAAATAG
Universal primers used for guide RNA synthesis (Burg et al., 2016)	
sgT7	GCTAGCTAATACGACTCACT
sgRNA-R	AAAAGCACCGACTCGGTG
M13F	GTAAAACGACGGCCAGT
Primers used to clone MO4-Tbx5a binding site<br>Yellow highlight: *tbx5a* 5′ UTR sequence<br>**Bold:** MO4-Tbx5a binding site<br>Green highlight: eGFP coding sequence	
tbx5aeGFP-F1	ttagatct**GCCTCACGGTAGACATCGTACAGGC**CTCTCCGACggatccatggtgagcaagggcg
tbx5aeGFP-F2	ttagatct**GCCTCACGGTAGACA**---**TACAGGC**CTCTCCGACggatccatggtgagcaagggcg
tbx5aeGFP-F3	ttagatct**GCCTCACGGTAGACA**--------**GGC**CTCTCCGACggatccatggtgagcaagggcg

Supplementary Table 1

MO (based on zfin.org)	Sequence 5′ to 3′	Reference
tbx5a-MO4	GCCTGTACGATGTCTACCGTGAGGC	tbx5-MO2 in Lu et al., 2008
ctnnb1-MO2	CTGGGTAGCCATGATTTTCTCACAG	β-catenin-1 MO1 in Bellipanni et al., 2006
ctnnb2-MO1	CCTTTAGCCTGAGCGACTTCCAAAC	β-catenin-2 MO2 in Bellipanni et al., 2006

Supplementary Table 2
